# Exploration of key genes associated with oxidative stress in polycystic ovary syndrome and experimental validation

**DOI:** 10.3389/fmed.2025.1493771

**Published:** 2025-02-27

**Authors:** Qinhua Li, Lei Liu, Yuhan Liu, Tingting Zheng, Ningjing Chen, Peiyao Du, Hong Ye

**Affiliations:** ^1^The First College of Clinical Medical Science, China Three Gorges University, Yichang, China; ^2^Department of Obstetrics and Gynecology, Yichang Central People’s Hospital, Yichang, China; ^3^Institute of Obstetrics and Gynecology, China Three Gorges University, Yichang, China; ^4^China Three Gorges University, Yichang, China; ^5^Central Laboratory, The First College of Clinical Medical Science, China Three Gorges University and Yichang Central People’s Hospital, Yichang, China; ^6^Hubei Key Laboratory of Tumor Microenvironment and Immunotherapy, China Three Gorges University, Yichang, China

**Keywords:** polycystic ovary syndrome, oxidative stress-related genes, machine learning, key genes, drug prediction

## Abstract

**Introduction:**

The current study demonstrated that oxidative stress (OS) is closely related to the pathogenesis of polycystic ovary syndrome (PCOS). However, there are numerous factors that lead to OS, therefore, identifying the key genes associated with PCOS that contribute to OS is crucial for elucidating the pathogenesis of PCOS and selecting appropriate treatment strategies.

**Methods:**

Four datasets (GSE95728, GSE106724, GSE138572, and GSE145296) were downloaded from the gene expression omnibus (GEO) database. GSE95728 and GSE106724 were combined to identify differentially expressed genes (DEGs) in PCOS. weighted gene correlation network analysis (WGCNA) was used to screen key module genes associated with PCOS. Differentially expressed OS related genes (DE-OSRGs) associated with PCOS were obtained by overlapping DEGs, key module genes, and OSRGs. Subsequently, the optimal machine model was obtained to identify key genes by comparing the performance of the random forest model (RF), support vector machine model (SVM), and generalized linear model (GLM). The molecular networks were constructed to reveal the non-coding regulatory mechanisms of key genes based on GSE138572 and GSE145296. The Drug-Gene Interaction Database (DGIdb) was used to predict the potential therapeutic agents of key genes for PCOS. Finally, the expression of key OSRGs was validated by RT-PCR.

**Results:**

In this study, 8 DE-OSRGs were identified. Based on the residuals and root mean square error of the three models, the best performance of RF was derived and 7 key genes (*TNFSF10, CBL, IFNG, CP, CASP8, APOA1*, and *DDIT3*) were identified. The GSEA enrichment analysis revealed that *TNFSF10, CP, DDIT3*, and *INFG* are all enriched in the NOD-like receptor signaling pathway and natural killer cell-mediated cytotoxicity pathways. The molecular regulatory network uncovered that both *TNFSF10* and *CBL* are regulated by non-coding RNAs. Additionally, 70 potential therapeutic drugs for PCOS were predicted, with ibuprofen associated with *DDIT3* and *IFNG*. RT-qPCR validation confirmed the expression trends of key genes *IFNG*, *DDIT3*, and *APOA1* were consistent with the dataset, and the observed differences were statistically significant (*P* < 0.05).

**Conclusion:**

The identification of seven key genes and molecular regulatory networks through bioinformatics analysis is of great significance for exploring the pathogenesis and therapeutic strategies of PCOS.

## 1 Introduction

PCOS is a common endocrine and metabolic disorder affecting women of reproductive age, with significant individual differences and an estimated prevalence between 6 and 15% (1). The diverse clinical manifestations of PCOS are currently diagnosed using the Rotterdam criteria established at the 2004 ESHRE/ASRM consensus workshop. The diagnose criteria include hyperandrogenemia or clinical manifestations of excessive androgens, oligo-ovulation, and the presence of polycystic ovaries, meeting at least two of the three criteria mentioned above and excluding other related conditions. Affected women may experience oligomenorrhea, irregular uterine bleeding, infertility, high rates of miscarriage, and an increased risk of endometrial hyperplasia and cancer. Additionally, women with PCOS face a higher risk of several serious health conditions, including diabetes, hypertension, lipid disorders, and metabolic syndrome. Moreover, they are more susceptible to mental health issues, such as depression, anxiety, bipolar disorder, obsessive-compulsive disorder, somatization, eating disorders, and reduced sexual satisfaction. Studies have also shown that women with PCOS are three to four times more likely to develop pregnancy-induced hypertension and preeclampsia, and twice as likely to have preterm birth compared to the general female population (2–5). Espite extensive research on PCOS, its etiology remains unclear, involving pathophysiological factors such as inflammation, endothelial dysfunction, hyperandrogenemia, hyperinsulinemia or insulin resistance (IR), oxidative stress (OS), and genetic mechanisms (6, 7). Clinical guidelines focus on lifestyle changes, including exercise and dietary modifications, as well as pharmacological treatments such as oral contraceptives (OCPs), metformin, and pioglitazone, to manage symptoms related to hyperandrogenemia and glucose intolerance (8). However, current treatment methods cannot cure the disease.

The role of OS in the pathogenesis of PCOS is increasingly receiving attention (7). OS is a physiological dysfunction resulting from the imbalance between free radicals produced during oxidation processes and the body’s ability to neutralize these free radicals, which is affected by certain pathological conditions. This imbalance has a complex relationship with IR, hyperandrogenemia, metabolic disorders, and disturbances in the gut microbiota (9). Many studies have shown that PCOS patients have significantly higher levels of circulating oxidative markers compared to healthy individuals (10, 11). Combating the risk of OS damage aids in the treatment of PCOS (12, 13). However, it is not clear which key genes are involved in the OS process in the development of PCOS. There is a relative lack of information on the molecular regulatory mechanisms of OS-related genes (OSRGs) in PCOS. Bioinformatics is an interdisciplinary field that combines biology, computer science, information technology, and mathematics, aiming to manage and analyze biomedical data (14). With the rapid development of high-throughput sequencing technologies and gene chip technologies, bioinformatics has become an important tool for understanding the molecular mechanisms of complex diseases, identifying disease biomarkers, and discovering new therapies (14). It is not only widely used in the analysis of genomic, transcriptomic, and proteomic data, but also plays a significant role in the study of disease mechanisms, drug discovery, and personalized medicine (15). In recent years, advancements in bioinformatics have enabled researchers to delve deeper into the molecular mechanisms associated with PCOS. For example, He et al. (16) studied the role of hub genes in the development of PCOS using bioinformatics methods and found that the hub gene PTER can significantly reduce the risk of PCOS. These studies provide new tools and methods for understanding PCOS, allowing researchers to explore the molecular mechanisms of this complex disease more comprehensively. The goal of this study is to identify OSRGs in PCOS and construct a competitive endogenous RNA (ceRNA) network centered on key OSRGs. Explore therapeutic drugs for PCOS based on key genes.

## 2 Materials and methods

### 2.1 Collecting and processing data

Four microarray datasets related to PCOS patients were downloaded from the GEO database.^1^ GSE95728 (platform: GPL16956) includes ovarian granulosa cells from 7 PCOS patients and 7 normal controls. GSE106724 (platform: GPL21096) includes ovarian granulosa cells from 4 PCOS patients and 4 normal controls. GSE138572 includes ovarian granulosa cells from 10 samples (PCOS: Control = 5:5), and the GSE145296 dataset includes cumulus cells from 12 samples (PCOS: Control = 6:6). These two datasets were used to construct the ceRNA regulatory network. RNA-SEQ sequencing was used for GSE138572, and microarray sequencing was used for GSE95728, GSE106724, and GSE145296. All four datasets used Rotterdam as the criterion to screen eligible patients. Patient clinical information was shown in Supplementary Table 1. OSRGs were obtained from the GeneCards database^2^ to identify genes associated with OS in PCOS, 375 OSRGs were downloaded by searching for “oxidative stress” and setting parameters with relevance scores > 25.

### 2.2 Data pre-processing and differential expression analysis

The samples from GSE95728 and GSE106724 were combined to eliminate the batch effect in the 22 case samples using the combat function of sva (v3.38.0) (17). In this process, the parameter dat represented the combined expression spectrum, batch indicated the batch information, and par.priority = TRUE was used to apply a parametric Bayesian approach. Subsequently, principal component analysis (PCA) was performed to explore the distribution of control and disease samples in the combined dataset. The parameters col = batch (grouping by batch) and pch = 16 (solid dots) were applied for visualization. Further, the DEGs were acquired between PCOS and controls in combined dataset via limma (v3.42.2) (18) (| log2FC| > 0.5 and *P* < 0.05). The volcano and heat maps of DEGs were generated with ggplot2 (v 3.4.1) (19) and pheatmap (v 1.0.12) package,^3^ respectively.

### 2.3 Construction of gene co-expression network

Weighted gene correlation network analysis (WGCNA) was used to identify gene modules with similar expression patterns and analyze their correlation with specific traits. We used WGCNA (v 1.69) (20) to construct a gene co-expression network for PCOS in combined dataset. First, the samples were clustered and outlier samples were eliminated. Then, a suitable soft threshold (β) was selected to construct a scale-free network based on the fact that the R2 exceeded 0.85 and the mean connectivity tended to zero. Subsequently, minModuleSize was set to 100, genes were categorized into different modules by the dynamic cutting tree algorithm. Further, the correlation between PCOS and modules was explored using Pearson, and modules with significant correlation were selected as key modules (*P* < 0.05). Key module genes were selected with | gene significance (GS)| > 0.8 and module membership (MM) > 0.8.

### 2.4 Enrichment analysis

DE-OSRGs significantly associated with PCOS were obtained by intersecting key module genes, DEGs, and OSRGs. Further, enrichment analysis was implemented using clusterProfiler (v3.18.0) to probe pathway and function related to DE-OSRGs, including Gene ontology (GO) and Kyoto Encyclopedia of Genes and Genome (KEGG). Among them, GO consists of 3 parts, namely biological processes (BP), molecular functions (MF) and cellular components (CC). The enrichment results were visualized using string plots with GO plot (v 1.0.2) (21) (Showing entries with gene number ≥ 2).

### 2.5 Machine learning

Machine learning was used to identify OSRGs, construct a ceRNA network, and explore potential therapeutic agents for PCOS based on OSRGs. In combined dataset, the caret (v 6.0-94) (22) was used to construct three models: random forest (RF), support vector model (SVM), and generalized linear model (GLM). Interpretative analysis of the three models was carried out using the explain function of DALEX (v2.4.3) (23) and the plot function was employed to visualize the distribution of model performance. Key genes for PCOS were filtered based on the cumulative residuals of samples and feature importance in each algorithm.

### 2.6 Establishment of molecular networks

In GSE138572, edgeR (v 3.38.4) (24) was exploited to filter for DE-miRNAs in disease and control samples (| log2FC| > 0.5 and *P* < 0.05) in GSE138572. Likewise, the DE-lncRNAs and DE-circRNAs were acquired between PCOS and controls using limma with | log2FC| > 0.5 and *P* < 0.05 in GSE95728 and GSE145296, respectively. Moreover, we constructed ceRNA networks to understand the regulatory mechanisms of key genes. To be consistent with the ceRNA hypothesis, the expression of key genes in PCOS and normal controls was obtained using the rank sum test. Targeted miRNA prediction of key genes was performed using miRWalk^4^ (Score = 0.95, Position = 3UTR) and the intersection was taken with DE-miRNAs. LncRNAs interacting with the intersecting miRNAs were predicted using the starBase database^5^ and then compared with the DE-lncRNAs in GSE95728. Similarly, the circRNAs interacting with the intersecting miRNAs were forecasted by circBank database^6^ and intersected with the DE-circRNA in GSE145296. The ceRNA network were constructed using Cytoscape (v 3.9.1) (25) software.

### 2.7 Gene set enrichment analysis (GSEA)

To further investigate the potential molecular mechanisms of key genes in PCOS, correlation analysis of key genes with all genes in PCOS patient samples was performed sequentially. Then, key genes were analyzed for GSEA enrichment by the Clusterprofiler (v 3.18.1) package (26) (*P*-value < 0.05).

### 2.8 Drug prediction

Potential drugs were predicted using the Drug-Gene Interaction Database (DGIdb)^7^ and visualized using Cytoscape software.

### 2.9 Reverse transcription quantitative real-time PCR (RT-qPCR)

The expression of these OSRGs in ovarian GCs from PCOS patients was validated by RT-qPCR to identify valuable OSRGs. A set of 6 pairs of control and PCOS samples were obtained from Yichang Central People’s Hospital. During *in vitro* fertilization-Embryo Transfer (IVF-ET), women aged 22 to 35 years with abnormal menstrual cycles were screened as PCOS patients. PCOS patients were diagnosed based on the 2003 Rotterdam Consensus, with no complications identified. Conditions such as Cushing’s syndrome, androgen-producing tumors, and congenital adrenal hyperplasia were excluded. The women met these criteria: (1) aged 22 to 35 years; (2) normal morphology and regular menstrual cycles (21–35 days); and (3) no tubal or male factor infertility during IVF-ET (control group).

Following the manufacturer’s protocol, total RNA was extracted from the 12 samples using TRIzol reagent (Invitrogen, China). After RNA concentration was measured using a NanoPhotometer N50, cDNA reverse transcription performed using the SureScript-First-strand-cDNA-synthesis-kit (Servicebio, China). Subsequently, 40 cycles were performed at 95°C for 1 min, denaturation at 95°C for 20 s, annealing at 55°C for 20 s, and extension at 72°C for 30 s. The relative quantification of mRNAs was computed by the 2^–Δ^ ^Δ^ *^CT^* method. The sequences of all primers could be found in Table 1.

**TABLE 1:** Primer details for reverse transcription quantitative polymerase chain reaction (RT-qPCR).

Primers	Sequences
TNFSF10 F	AGTCAAGTGGCAACTCCGTC
TNFSF10 R	GAGCTGCTACTCTCTGAGGAC
CBL F	AGGGAAAGCATGAGACTGGC
CBL R	CTTTGGAGCTCTCACTGCCA
IFNG F	TGCAATCTGAGCCAGTGCTT
IFNG R	GCACCAGGCATGAAATCTCC
CASP8 F	CTGAGCTGGTCTGAAGGCTG
CASP8 R	GGGTTCTTGCTTCCTTTGCG
DDIT3 F	TTCTCTGGCTTGGCTGACTG
DDIT3 R	TTCCTGCTTGAGCCGTTCAT
APOA1 F	GAGACTGCGAGAAGGAGGTC
APOA1 R	TCTCTGCCGCTGTCTTTGAG
internal reference R-GAPDH F	GACCCCTTCATTGACCTCAAC
internal reference R-GAPDH R	GCCATCACGCCACAGCTTTCC

### 2.10 Ethics approval

All participants provided informed consent, and the study received approval from the Hospital of ethics IRB, Approval Number: AF/SC-09/1.0.

### 2.11 Statistical analysis

This study was all conducted in R language. If not specified, *P* < 0.05 indicated statistical significance.

## 3 Results

### 3.1 Identification of DEGs in PCOS

**FIGURE 1:**
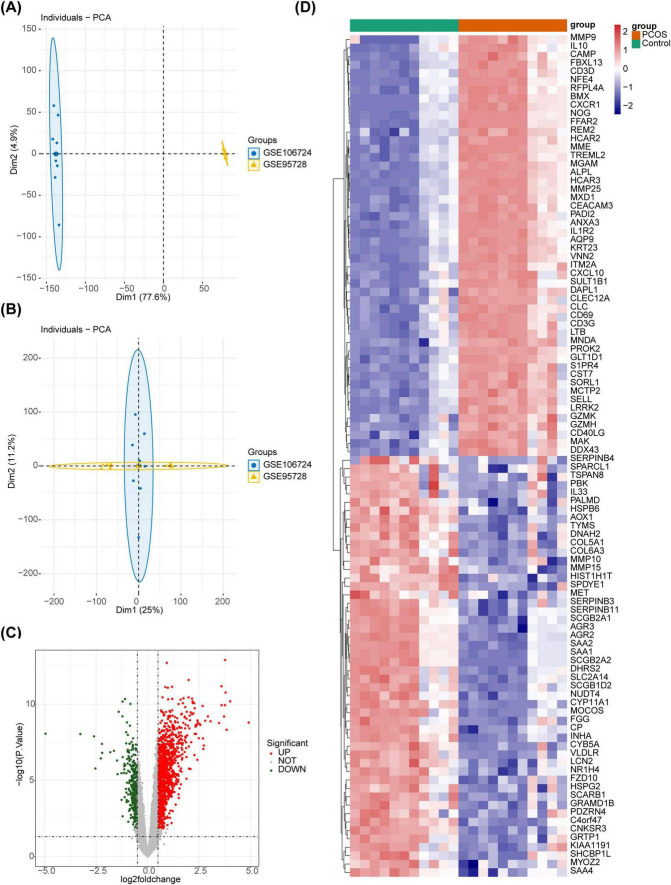
The DEGs between the PCOS and the control groups. **(A)** The sample distribution before batch effect treatment. **(B)** The sample distribution after batch effect treatment. **(C)** The volcano plot of differential gene analysis. Red dots represent upregulated genes, green dots represent downregulated genes, and gray dots represent genes with no significant difference, or small fold changes. **(D)** The heatmap of differential gene analysis.

After eliminated the differences between batches of 22 samples, the PCA showed that the different batches were at the same level after correction, and there were significant differences before and after batch treatment (Figures 1A, B). Further, the 1,514 DEGs were acquired between PCOS and controls, of which 1,149 were enhanced and 365 were educed (Figures 1C, D).

### 3.2 Identification of key module genes for PCOS

**FIGURE 2:**
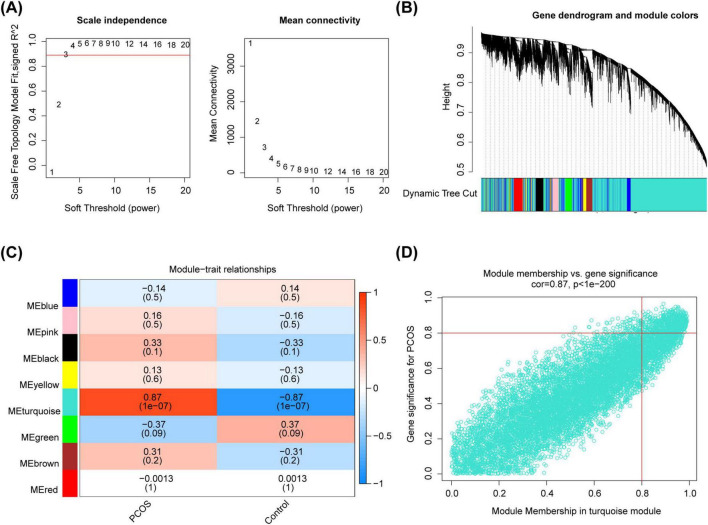
The identification of key module genes. **(A)** The choosing soft threshold. **(B)** The division of module genes. **(C)** The heat map of correlation between modules and traits. **(D)** The key genes of the turquoise module.

In the integrated dataset, samples were clustered and there were no outliers (Supplementary Figure 1). Choosing Soft Threshold 3 to Build Scale-Free Networks (R^2^ = 0.89) (Figure 2A). Then, 8 module genes were obtained (Figure 2B), among which the turquoise module had a remarkable strong correlation with PCOS as the key module (cor = 0.87, *P* = 1 × 10^–7^) (Figure 2C). Based on | GS| > 0.8 and MM > 0.8, 623 key module genes were acquired (Figure 2D).

### 3.3 Pathways and functions involved in DE-OSRGs

**FIGURE 3:**
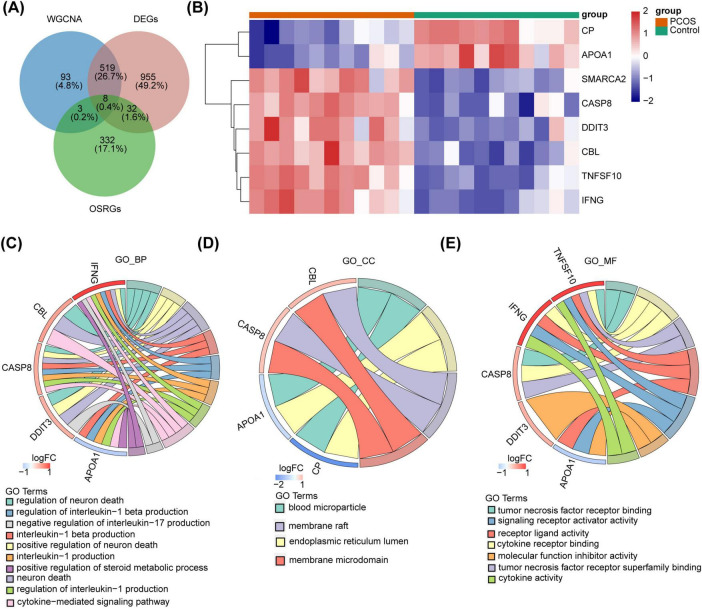
The DE-OSRGs identification and its enrichment analysis. **(A)** The Venn diagram of DEGs, key module genes, and OSRGs. **(B)** The heatmap of expression in the PCOS and control groups. **(C–E)** The GO functional enrichment analysis of DE-OSRGs.

The DEGs, key module genes, and OSRGs were intersected to gain 8 DE-OSRGs (Figure 3A). Figure 3B demonstrated 8 DE-OSRGs in PCOS patients and normal controls. Moreover, these genes were enriched 461 GO terms, which yielded 388 BPs, such as regulation of neuron death, cytokine-mediated signaling pathway, regulation of interleukin-1 production, 26 CCs, such as endoplasmic reticulum lumen, membrane microdomain, blood microparticle, membrane raft, 47 MFs, cytokine receptor binding, signaling receptor activator activity (Figures 3C–E and Supplementary Table 2).

### 3.4 Identification of key genes

**FIGURE 4:**
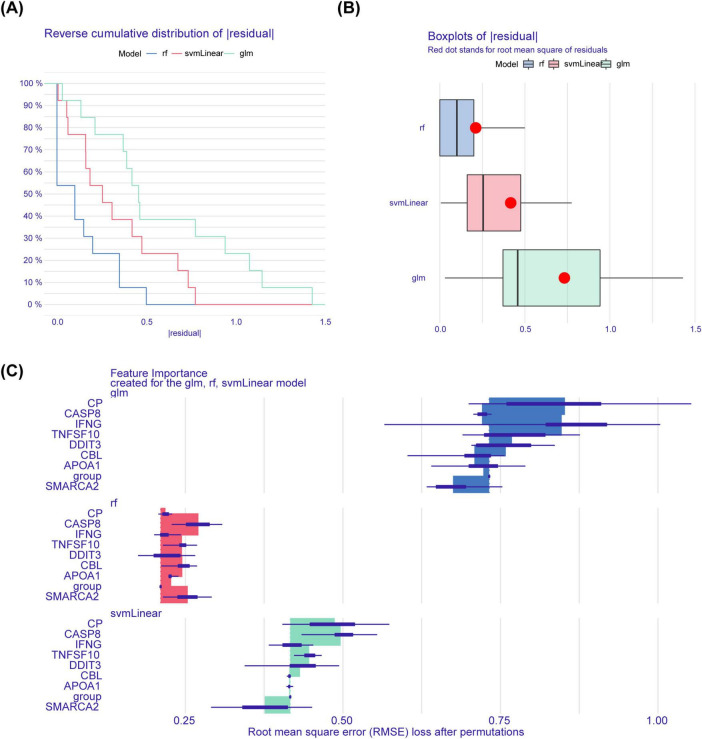
The identify key genes for PCOS. **(A)** The plot of cumulative residual distribution for the sample. **(B)** The box plots of sample residuals. **(C)** The importance of variables in RF, GLM, and SVM models.

To identify key genes for PCOS, we constructed three machine-learning models (RF, SVM, GLM) based on 8 DE-OSRGs. Further, the RF model had the smallest sample residuals and was treated as the optimal model (Figures 4A, B). Moreover, TNF superfamily member 10 (TNFSF10), Cbl proto-oncogene (CBL), Interferon gamma (IFNG), Ceruloplasmin (CP), Caspase 8 (CASP8), Apolipoprotein A-I (APOA1) and DNA damage inducible transcript 3 (DDIT3) had greater effect on predictive value of the response variables, and were therefore considered as key genes for PCOS (Figure 4C).

### 3.5 Construction of ceRNA regulatory network based on key genes

In GSE138572, there were 35 DE-miRNAs, including 24 increased miRNAs and 11 decreased miRNAs. In GSE95728, there were 641 DE-lncRNAs, including 325 elevated lncRNAs and 316 diminished lncRNAs. In GSE145296, there were 4,532 DE-circRNAs, of which 2,364 were enhanced circRNAs and 2,168 were reduced circRNAs. The volcano plots of DE-miRNAs, DE-lncRNAs, and DE-circRNAs were shown in Supplementary Figure 2.

**FIGURE 5:**
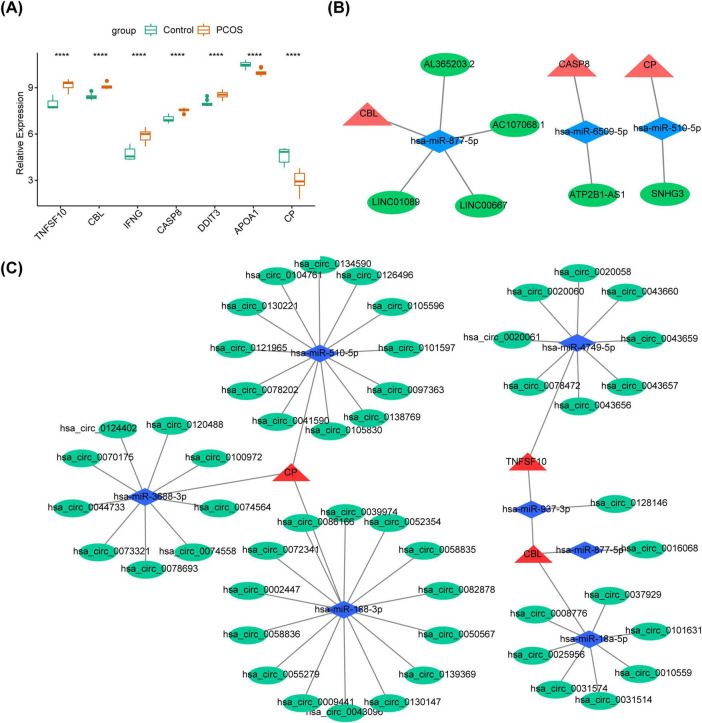
The construction of ceRNA regulatory network. **(A)** The polycystic ovary syndrome (PCOS) and the control groups difference miRNA volcano plot ****P < 0.05. **(B)** The polycystic ovary syndrome (PCOS) and the control groups difference lncRNA volcano plot. **(C)** The polycystic ovary syndrome (PCOS) and the control groups difference circRNA volcano plot.

Further, TNFSF10, CBL, IFNG, CASP8 and DDIT3 were remarkably up-regulated in the PCOS group, while APOA1 and CP were markedly down-regulated (*P* < 0.05) (Figure 5A). Based on 5 up-regulated genes, 1,447 miRNAs were predicted, overlapping with 11 down-regulated miRNAs to obtain 5 target miRNAs (Supplementary Figure 3A). Then, 218 lncRNAs with interactions with target miRNAs were predicted, overlapping with 120 up-regulated lncRNAs to obtain 5 target lncRNAs (Supplementary Figure 3B). Similarly, based on 2 down-regulated genes, 4 target miRNAs (Supplementary Figure 3C) and 1 targeted lncRNA (Supplementary Figure 3D). Subsequently, these results were integrated to construct lncRNA-miRNA-mRNA network containing 12 points and 9 edges (Figure 5B). CBL was found to be regulated by hsa-miR-877-5p and multiple long non-coding RNAs, including AC107068.1 and LINC00667. CASP8 was observed to be regulated by hsa-miR-6509-5p and ATP2B1-AS1. CP was identified as being regulated by hsa-miR-510-5p and SNHG3. Additionally, based on 5 up-regulated genes, 5 targeted miRNAs (Supplementary Figure 3E) and 17 targeted circRNAs (Supplementary Figure 3F) were obtained. The 4 targeted miRNAs (Supplementary Figure 3G) and 35 targeted circRNAs (Supplementary Figure 3H) were acquired based on 2 down-regulated genes. Subsequently, the data were integrated and an mRNA-miRNA-circRNA network was constructed, which contained 62 points and 60 edges (Figure 5C). We found that CP were all regulated by hsa-miR-3688-3p, hsa-miR-188-3p and hsa-miR-510-5p as well as a large number of circRNAs, such as hsa_circ_0105830, hsa_circ_0043096, hsa_circ_0074564.

### 3.6 Pathways involved in key genes

**FIGURE 6:**
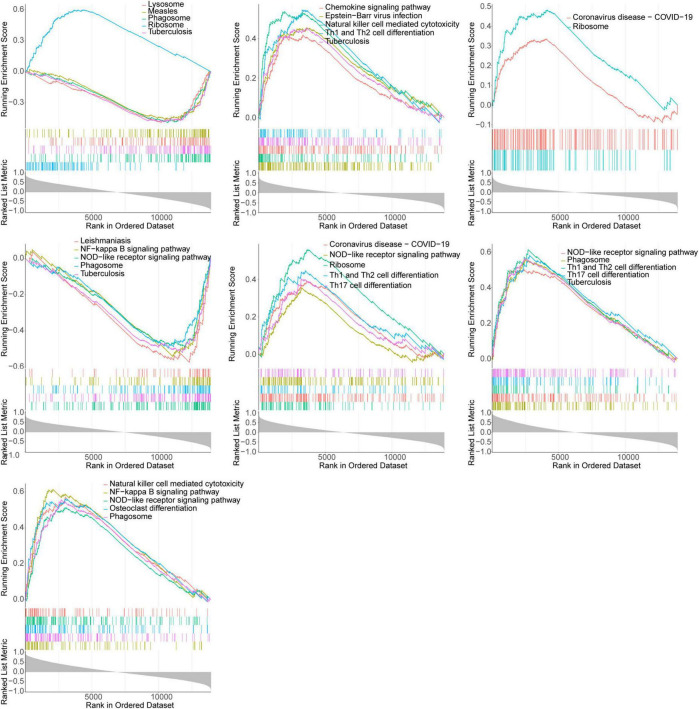
GSEA enrichment analysis of key genes showed significant enrichment of the top 5 KEGG pathways (APOA1, IFNG, CBL, CP, CASP8, DDIT3, and TNFSF10 in gene order).

We further explored the pathways involved in the key genes. The Figure 6 demonstrated the KEGG pathway of TOP5 in which each gene was involved (SIZE > 10 and *P*-value < 0.05). All other pathways were shown in Supplementary Tables 3–9. The GSEA enrichment analysis showed that TNFSF10, CP, *DDIT3* and *INFG* were all enriched in NOD-like receptor signaling pathway, NLR signaling pathways, NK cell-mediated cytotoxicity and NF-kappa B signaling pathway. Furthermore, *CASP8*, *APOA1*, CP and INFG were all enriched in tuberculosis (Figure 6).

### 3.7 Key gene-based prediction of potential therapeutic agents

**FIGURE 7:**
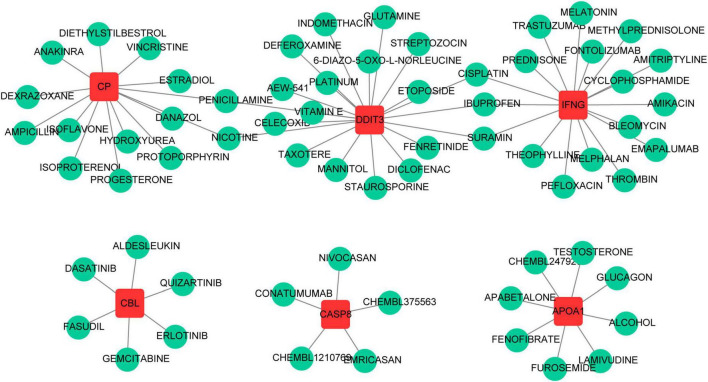
The prediction of potential therapeutic drugs for key genes.

A total of 70 potential drugs for the treatment of PCOS were predicted using the DGIdb database, based on the analysis of 7 key genes (Supplementary Table 10). Of these, penicillamine and nicotine were both associated with *CP* and *DDIT3*, and suramin, ibuprofen and cisplatin were all linked to *IFNG* and *DDIT3* (Figure 7).

### 3.8 Expression of key genes in clinical samples

**FIGURE 8:**
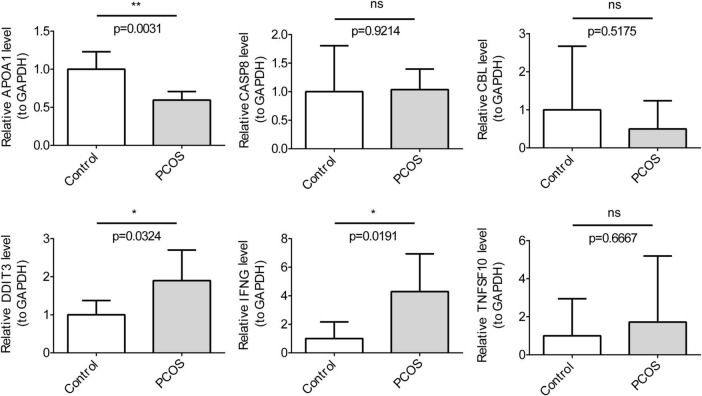
The qRT-PCR clinical sample Validation for key genes (*n* = 3). The *t*-tests were performed for statistical analysis.*P < 0.05, **P < 0.01.

We examined the expression levels of key genes in clinical samples using RT-qPCR. *TNFSF10*, *IFNG*, *CASP8*, and *DDIT3* all exhibited high expression in the disease group, while *CBL* and *APOA1* presented low expression (Figure 8). This was consistent with the expression trend in the dataset. In particular, there were significant differences in gene expression between the two groups for *IFNG*, *DD1T3*, and *APOA1* (*P* < 0.05). Subsequent clinical samples could be expanded to test the expression of key genes.

## 4 Discussion

PCOS is a common endocrine and metabolic disorder in women. Despite progress in the treatment of PCOS over the past decades, the pathogenesis of the disease remains unclear due to its high heterogeneity, and current therapies are unable to cure the disease (5, 6). Therefore, it is crucial to delve into the causes of PCOS. OS is related to the pathophysiology of PCOS (7). Numerous studies have shown that the levels of OS markers in the serum and follicular fluid of PCOS women are elevated, which may adversely affect follicle growth, oocyte maturation, and embryo quality, ultimately leading to infertility (27). IR, hyperandrogenemia, metabolic disorders, and gut microbiota are associated with OS (11). The increased OS subsequently leads to the production of pro-inflammatory cytokines (7), which may induce IR and hyperandrogenemia, and increase the risk of cardiovascular diseases (28, 29). Although there is substantial evidence that OS significantly affects the progression of PCOS, which OSRGs exist in PCOS and their molecular regulatory mechanisms are not clear.

In this study, four datasets from the GEO database—GSE95728, GSE106724, GSE138572 (miRNA), and GSE145296 (circRNA)—were subjected to bioinformatics analysis to identify genes related to OS in PCOS. The results indicated that TNFSF10, CBL, IFNG, CP, CASP8, APOA1, and DDIT3 may be associated with the pathogenesis of PCOS. However, in our analysis, possibly due to data limitations and analytical method factors, we did not find that estradiol, progesterone, and testosterone are key OSRGs. We validated the OSRGs in GCs from PCOS patients. Compared to normal control group patients, IFNG, TNFSF10, and DDIT3 were highly expressed in GCs from PCOS patients, while CBL and APOA1 were lowly expressed.

IFNG is a gene encoding a member of the cysteine-aspartic protease (caspase) family and is involved in programmed cell death induced by Fas and various apoptotic stimuli. IFNG drives CASP8/FADD-mediated necroptosis and pyroptosis by altering the levels of the oxidative stress marker NO (30). Studies have shown that IFNG is present in ovarian follicular fluid and lymphocytes and is an important regulatory factor for the differentiation of GCs during follicular development. In women with PCOS-related infertility, IFNG levels in peripheral blood and follicles are significantly higher than in normal women (31, 32), and the incidence of granulosa cell apoptosis in women with PCOS is higher than in the control group (33). *In vitro* experiments have shown that IFNG promotes apoptosis in the human granulosa-like tumor cell line (KGN) cells (34). IFNG stimulates IGFBPs to reduce the activity of endogenous insulin-like growth factor (IGF) and promotes apoptosis of luteinized granulosa cells, thereby facilitating luteal regression (35). IFNG produced by adipose tissue inhibits adipose tissue homeostasis (36), leading to insulin resistance (IR) and metabolic syndrome, and reducing IFNG levels can improve IR and metabolic syndrome (37). Metformin can reduce IFNG levels, inhibit pyroptosis in ovarian macrophages, and improve PCOS (38). These findings suggest that IFNG may be involved in the pathophysiological process of PCOS by promoting apoptosis of ovarian GCs and IR.

DDIT3, also known as transcription factor C/EBP homologous protein (CHOP), is an endoplasmic reticulum (ER) stress effector (39). ER stress is a local factor closely associated with inflammation and OS. When ER stress is severe and persistent, the unfolded protein response (UPR) may lead to upregulation of DDIT3 expression (40). DDIT3 is significantly elevated in the granulosa cells (GCs) of women with PCOS and in a DHEA-induced PCOS mouse model (41). ER stress in GCs of women with PCOS promotes ovarian tissue fibrosis (42). DDIT3 can regulate the expression of TNFRSF10B (also known as DR5), upregulate the expression of TNFRSF10B, and mediate granulosa cell apoptosis. In human granulosa lutein cells (GLCs) pre-cultured with testosterone, the expression of death receptor (DR) 5 and DDIT3 is upregulated. Testosterone induces the upregulation of DR5 by inducing CHOP, leading to apoptosis in human GLCs (41). Apoptosis of ovarian granulosa cells can trigger follicular atresia, which may significantly contribute to the development of PCOS (43–45). In summary, DDIT3 may be involved in the development of PCOS by affecting ovarian tissue fibrosis and granulosa cell apoptosis through ER stress.

APOA1 encodes apolipoprotein A-I, which is the main protein component of high-density lipoprotein (HDL) in plasma. APOA1 can protect immune cell membranes, circulating oxidized lipoproteins, and mitochondria from lipid peroxidation and oxidative damage (46, 47). A randomized controlled trial has shown that patients with PCOS have lower levels of APOA1 (48). An increase in the apolipoprotein B (APOB) to APOA1 ratio is associated with the worsening of metabolic syndrome (MetS) components in PCOS, including IR, elevated androgen levels, and increased liver enzyme levels (49). Chronically elevated levels of reactive oxygen species (ROS) and reactive nitrogen species (RNS) can lead to decreased levels of APOA1 in circulation (46), and chronic OS can induce dysfunction of APOA1 (50–52). ApoA1 plays an important role in antioxidant stress and immune regulation, but the local significance of decreased APOA1 expression in the ovaries of women with PCOS is still unclear and requires further research.

CBL is a RING domain-based E3 ubiquitin ligase that can transfer ubiquitin to substrates and accelerate protein degradation. In recent years, an increasing number of studies have emphasized the role of CBL in the regulation of OS (53, 54). Shen et al. (55) found through *in vitro* and animal studies that alterations in the phosphorylation and activity of CBL play a key role in the pathogenesis of PCOS and are closely related to insulin resistance IR in patients with PCOS. In addition, phosphorylated CBL protein can regulate downstream signaling cascades associated with PCOS and related IR, participate in oxidative stress (56), and is associated with inflammation of GCs (57).

TNFSF10 also known as TRAIL (TNF-related apoptosis-inducing ligand), a cytokine that induces apoptosis by binding to its corresponding receptors. This binding event initiates a cascade that leads to the activation of MAPK8/JNK, CASP8, and caspase 3, thereby triggering the apoptotic process (58). TNFSF10 induces OS in various cell types (59, 60). Wang et al. (61) found that TNFSF10 is overexpressed in GCs of PCOS patients and in ovarian GCs of a DHEA-induced PCOS rat model compared to the normal control group. Our RT-qPCR analysis also showed that TNFSF10 expression was increased in luteinized GCs from women with PCOS-related infertility compared with those without PCOS, although the difference was not statistically significant. TNFSF10 plays a role in regulating granulosa cell apoptosis in a sodium pregnanone sulfate-induced PCOS rat model (62), and the rate of granulosa cell apoptosis is associated with follicular development stagnation.

CASP8 is a crucial cysteine protease that primarily initiates the apoptosis process through the activation of death receptors, such as Fas and TNF receptors. One characteristic of PCOS is the presence of cystic follicles at various stages of growth and atresia, which is a result of apoptosis and tissue remodeling. Studies have shown that under conditions similar to PCOS induced by DHEA, the Fas/FasL/CASP8 (death receptor-dependent) pathway is activated, leading to apoptosis of ovarian GCs, which is crucial for follicular atresia (63). TNFSF10 initiates a series of cascades upon binding to its receptors, leading to the activation of CASP8, thereby triggering the apoptosis process (58). IFNG can drive the formation of the CASP8/FADD complex by altering the levels of the OS arker nitric oxide (NO) (30). These finding suggest that CASP8 may be involved in the development and progression of PCOS.

In addition, except for the CBL gene, the other six genes were enriched in the NOD-like receptor signaling (NLRs) and natural killer cell-mediated cytotoxicity pathways. TNFSF10, IFNG, CP, CASP8, and APOA1 were enriched in the NF-κB signaling pathway. The NLRs pathway and/or the NF-κB pathway control genes involved in inflammation and antioxidant processes, enhance inflammatory responses, and contribute to the development of PCOS (64–66). NK cells influence the progression of PCOS by secreting various cytokines (33, 67), inducing apoptosis (62), and participating in OS (68). Combining the existing research findings and RT-qPCR experimental validation from clinical samples, we have found that although the specific roles of the key genes identified in this study in relation to PCOS require further verification, these genes, whether directly or indirectly, are likely associated with the occurrence of PCOS (Supplementary Figure 4). This suggests that it is worthwhile to continue in-depth research on how these genes regulate the pathological processes of PCOS, providing new clues for the study of PCOS biomarkers.

CeRNA networks that regulate these OSRGs. LncRNAs, miRNAs and circRNA, non-coding RNAs (69), interact with each other and with mRNAs to regulate gene expression (70, 71), affecting processes like cholesterol production (72),IR (73), and cell proliferation (74), participating in the development process of PCOS. Our results show that CP, CBL, and TNFSF are mutually regulated and influenced by miRNAs and circRNAs. hsa-miR-937-3p regulates the expression of both CBL and TNFSF simultaneously. Hsa-miR-937-3p apparently improves the overall survival rate of patients with lung adenocarcinoma (75). In anti-tumor therapy, CBL can influence the anti-tumor functionality of TNFSF (76, 77). However, the specific roles of these molecules in PCOS have not been extensively reported and further research is needed.

In this study, we utilized the DGIdb to forecast compounds that correspond with seven pivotal genes. Our analysis led to the identification of several compounds, such as melatonin, ibuprofen, and vitamin E, which have been previously investigated for their potential antioxidants for the treatment of PCOS (78, 79). Ibuprofen can regulate the expression of DDIT3 and INFG. Additionally, Ibuprofen has been shown to reduce peripheral blood testosterone levels in PCOS patients (79). Melatonin may protect PCOS GCs from damage and improve ovarian dysfunction in PCOS models by regulating autophagy, reducing inflammation, and inhibiting apoptosis (80, 81). Supplementing with vitamin E can significantly reduce serum total testosterone levels in PCOS patients (82). Melatonin and vitamin E may be safe and useful supplements for improving PCOS. These results provide new compound clues for the treatment of PCOS. For example, for PCOS patients with high testosterone levels and mild metabolic abnormalities, the combined use of ibuprofen and vitamin E may help alleviate symptoms and improve metabolic conditions. For PCOS patients with sleep disorders, melatonin may be an effective treatment option (78). Of course, further verification of the efficacy and safety of these compounds in PCOS treatment is needed through relevant clinical trials and data analysis. Overall, these results expand the treatment options for PCOS and also help in the development of more personalized treatment strategies.

It is worth noting that there are some shortcomings in this study. Differences in sample sources and date limitations may lead to bias in the analysis results. Secondly, the specific mechanism of OS signature genes in PCOS needs to be further verified by *in vivo* and *in vitro* experiments in future studies.

## 5 Conclusion

Our study predicts the potential impact of OS on the progression of PCOS and identifies seven OS signature genes that may serve as biomarkers and therapeutic targets for PCOS patients. These findings provide novel insights into the pathophysiological mechanisms of the condition and suggest potential avenues for targeted therapeutic interventions.

## Data Availability

The datasets presented in this study can be found in online repositories. The names of the repository/repositories and accession number(s) can be found in this article/Supplementary material.

## Footnotes

1
https://www.ncbi.nlm.nih.gov/geo/

2
https://www.genecards.org/

3
https://cran.r-project.org/web/packages/pheatmap/index.html

4
http://mirwalk.umm.uni-heidelberg.de/

5
https://rnasysu.com/encori/

6
http://www.circbank.cn/help.html

7
https://beta.dgidb.org/
