# Supplementary material for: Exploration of key genes associated with oxidative stress in polycystic ovary syndrome and experimental validation
Source: Front Med (Lausanne). 2025 Feb 27;12:1493771. doi: 10.3389/fmed.2025.1493771 (PMC11904916; doi:10.3389/fmed.2025.1493771)
Supplement: Supplementary file 11 [file Presentation_1.pdf]

frontiers

Supplementary Material

1 Supplementary Figures and Tables

1.1 Supplementary Figures

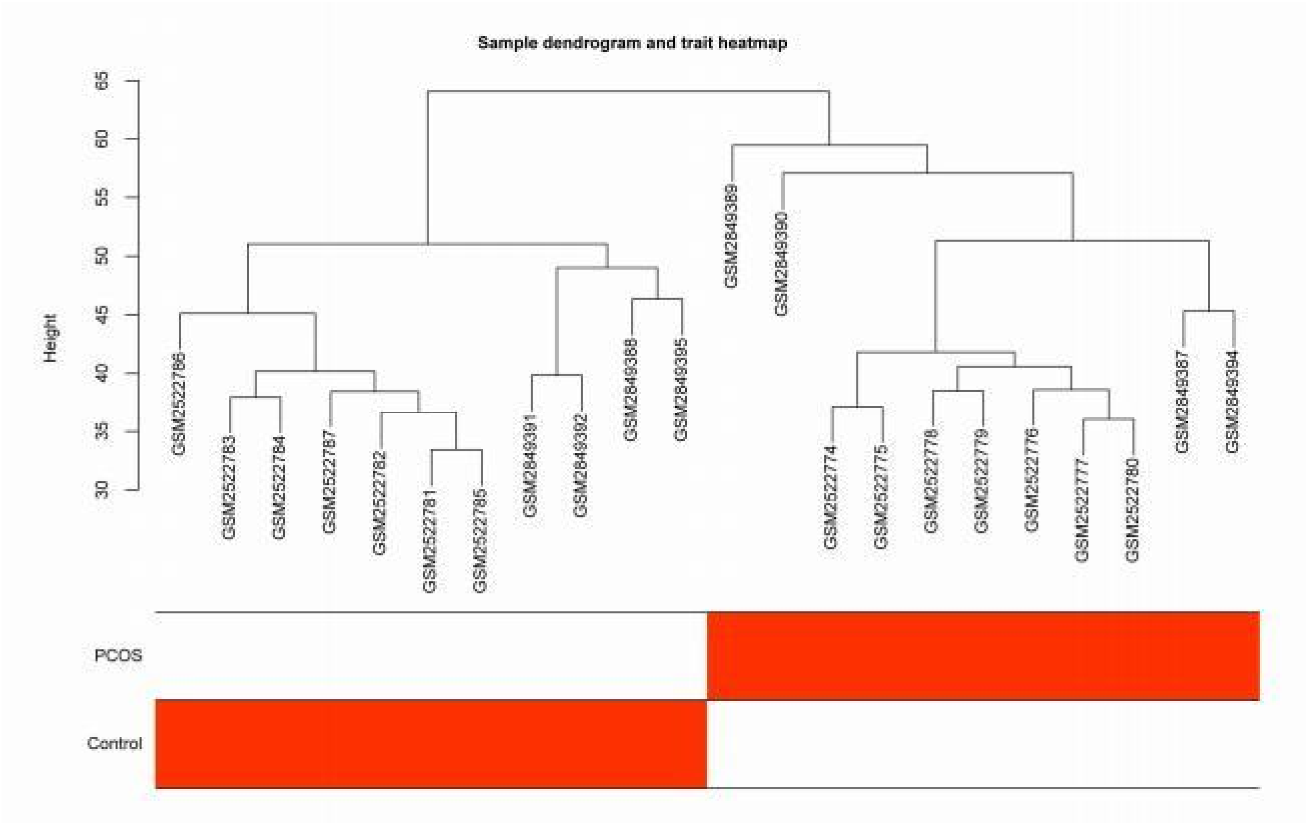

Supplementary Figure 1. The clustered and trait heatmaps of polycystic ovary syndrome (PCOS) and Control samples.

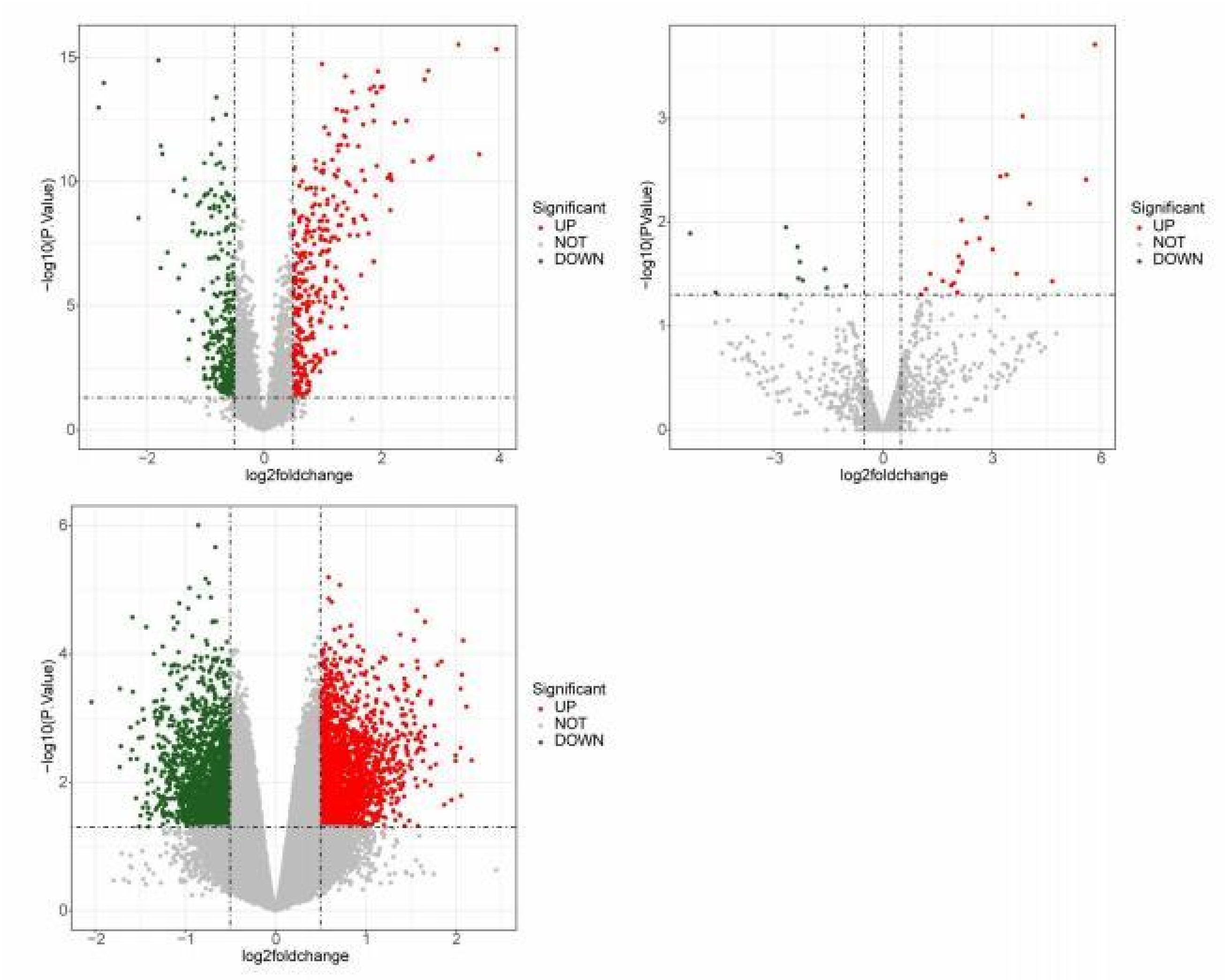

Supplementary Figure 2. The in GSE138572, volcano plots of DE-miRNAs, DE-lncRNAs, and DE-circRNAs

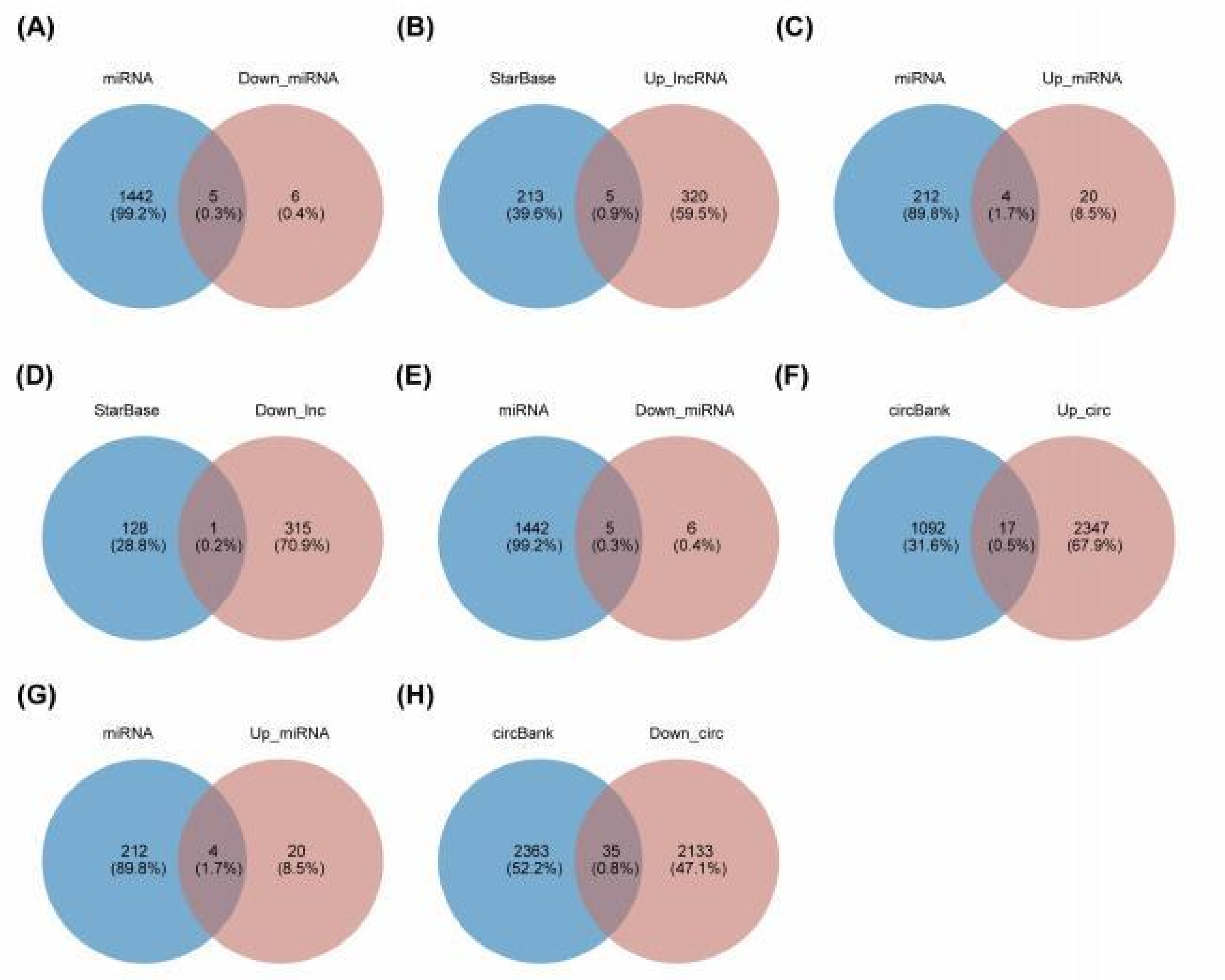

Supplementary Figure 3. The construction of the ceRNA regulatory network. **(A)** The venn diagram of miRWalk and Down_miRNA. **(B)** The venn diagram of StarBase and Up_lncRNA. **(C)** The venn diagram of miRWalk and Up_miRNA. **(D)** The venn diagram of StarBase and Down_lncRNA. **(E)** The venn diagram of miRNA and Down_miRNA. **(F)** The venn diagram of circBank and Up_circ. **(G)** The venn diagram of miRNA and Up_miRNA. **(H)** The mRNA-miRNA-circRNA regulatory network.

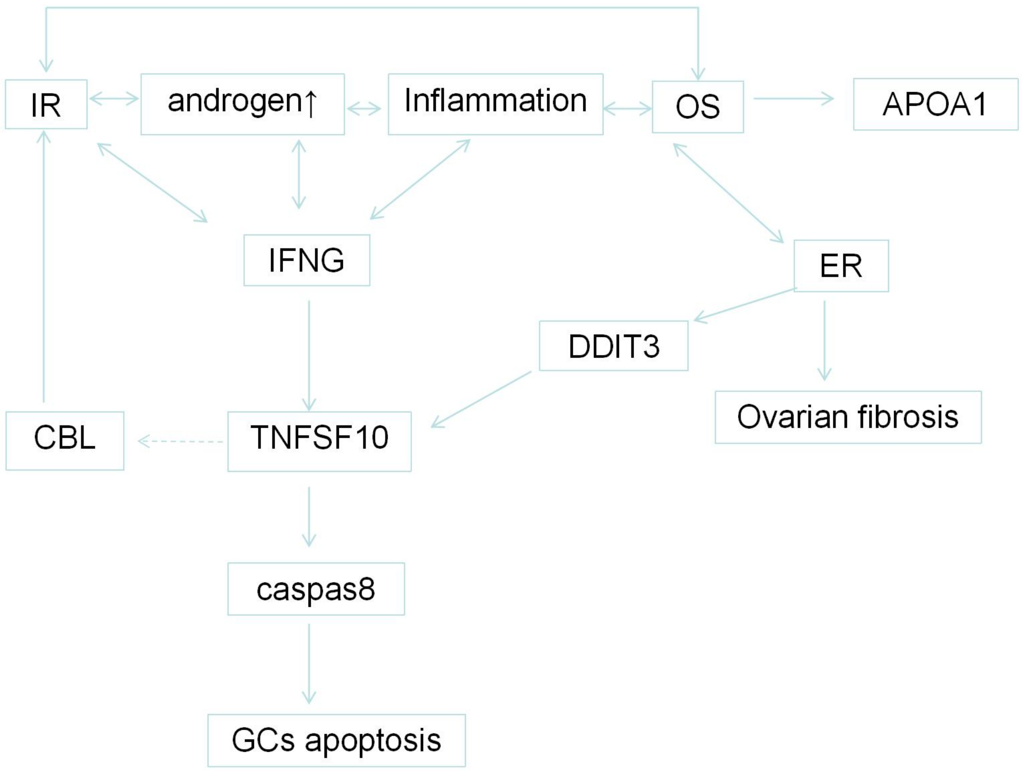

Supplementary Figure 4. Visual Summary. Light green solid arrows represent pathways that have been experimentally proven. Light green dashed arrows indicate a lack of experiments related to PCOS.

1.2 Supplementary Tables

Supplementary Table 1. Patient clinical information

Supplementary Table 2. The Gene Ontology (GO) functional enrichment analysis BP, CC and MF results of differentially expressed-OSRGs (DE-OSRGs).

Supplementary Table 3. The gene set enrichment analysis (GSEA) enrichment analysis of APOA1 .

Supplementary Table 4. The gene set enrichment analysis (GSEA) enrichment analysis of CASP8.

Supplementary Table 5. The gene set enrichment analysis (GSEA) enrichment analysis of CBL.

Supplementary Table 6. The gene set enrichment analysis (GSEA) enrichment analysis of CP.

Supplementary Table 7. The gene set enrichment analysis (GSEA) enrichment analysis of DDIT3.

Supplementary Table 8. The gene set enrichment analysis (GSEA) enrichment analysis of IFNG.

Supplementary Table 9. The gene set enrichment analysis (GSEA) enrichment analysis of TNFSF10.

Supplementary Table 10. The total of 70 potential drugs for the treatment.
